# Autologous iPSC-derived dopamine neuron transplantation in a nonhuman primate Parkinson’s disease model

**DOI:** 10.1038/celldisc.2015.12

**Published:** 2015-05-26

**Authors:** Shuyan Wang, Chunlin Zou, Linlin Fu, Bin Wang, Jing An, Gongru Song, Jianyu Wu, Xihe Tang, Mo Li, Jian Zhang, Feng Yue, Chengyun Zheng, Piu Chan, Y Alex Zhang, Zhiguo Chen

**Affiliations:** 1 Cell Therapy Center, Beijing Institute of Geriatrics, Xuanwu Hospital, Capital Medical University, and Key Laboratory of Neurodegeneration, Ministry of Education, Beijing, China; 2 Center of Neural Injury and Repair, Beijing Institute for Brain Disorders, Beijing, China; 3 Center of Parkinson's Disease, Beijing Institute for Brain Disorders, Beijing, China; 4 Center for Translational Medicine, Guangxi Medical University, Nanning, China; 5 Department of Neurobiology, Beijing Institute of Geriatrics, Xuanwu Hosptial, Capital Medical University, Beijing, China; 6 Department of Hematology, Second Hospital of Shandong University, Jinan, China

**Keywords:** iPSC, dopamine neurons, autologous, transplantation, Parkinson’s disease, cynomolgus monkey

## Abstract

Autologous dopamine (DA) neurons are a new cell source for replacement therapy of Parkinson’s disease (PD). In this study, we tested the safety and efficacy of autologous induced pluripotent stem cell (iPSC)-derived DA cells for treatment of a cynomolgus monkey PD model. Monkey bone marrow mesenchymal cells were isolated and induced to iPSCs, followed by differentiation into DA cells using a method with high efficiency. Autologous DA cells were introduced into the brain of a cynomolgus monkey PD model without immunosuppression; three PD monkeys that had received no grafts served as controls. The PD monkey that had received autologous grafts experienced behavioral improvement compared with that of controls. Histological analysis revealed no overgrowth of grafts and a significant number of surviving A9 region-specific graft-derived DA neurons. The study provided a proof-of-principle to employ iPSC-derived autologous DA cells for PD treatment using a nonhuman primate PD model.

## Introduction

Parkinson’s disease (PD) is a common neurodegenerative disease in aging population [1]. The clinical symptoms of PD include resting tremor, bradykinesia, muscle rigidity, and postural instability. The pathological feature of PD is the progressive neurodegeneration of dopaminergic (DA) neurons at the substantial nigra in midbrain. The lost cells in PD are restricted by cell type—only DA neurons are damaged, and by space—only at the midbrain; this feature makes PD an ideal disease candidate to receive cell therapy.

In 1990s, scientists have attempted to employ fetal ventral mesencephalon tissues to treat PD patients [2]. However, the two placebo-controlled double-blind clinical trials using fetal ventral mesencephalon tissues failed to meet the primary end points and only showed mild outcome [3, 4]. A careful retrospective analysis revealed several possible reasons for the mild outcome, which include immune recognition of the incoming grafts, heterogeneity of grafts [5], lack of standardization for the preparation of grafts and operation procedures, and suboptimal selection of patients.

The advent of induced pluripotent stem cell (iPSC) technology offers an opportunity to solve the above problems associated with fetal ventral mesencephalon transplants [6–8]. iPSCs possess identical or almost identical genetic background, and thus could greatly reduce the adverse effects of immune recognition [9]. The extensively proliferative capacity of iPSCs may meet the scalability requirements for cell therapy and provide sufficient base number of starter cells for differentiation and subsequent sorting process [10–12]. In 2013, Emborg and colleagues tested the concept of autologous transplantation of iPSC-derived DA neurons in a rhesus monkey PD model. However, with the differentiation scheme used in that study, very few graft-derived TH-positive neurons were detected, and no improvement in motor function was observed 6 months following autologous transplantation [13], implying that an improved differentiation method may be the key for good engraftment outcome. In the present study, we employed a different method for DA neuron specification and examined the safety and efficacy of autologous iPSC-derived DA cell transplantation in a cynomolgus PD model.

## Results

### iPSC derivation and characterization

Previous study shows that up to 100% of captive and free-ranging Asian nonhuman primate over 3 years old are infected with simian foamy viruses (SFV) [14]. SFV-positive cynomolgus monkeys remain asymptomatic [14, 15], but the virus interferes with iPSC induction *in vitro* (data not shown). By constant screening, we found one monkey that was SFV-negative and used it for the autologous transplantation experiment. Mesenchymal stem cells (MSCs) of this monkey were extracted from the bone marrow and expanded in culture. By using a classical retroviral transfection method [6, 7], MSCs were induced to iPSCs (Supplementary Figures S1A and B). The iPSCs were characterized and shown to be positive for alkaline phosphatase (AP), Nanog, Oct-4, SSEA-4, TRA-1-60, and TRA-1-81 (Supplementary Figure S1C). The iPSCs also showed normal karyotype (Supplementary Figure S1D) and the potential to form embyoid bodies and differentiate to cells of the three germ layers (Supplementary Figure S1E).

### Differentiation of iPSCs to DA neurons

**Figure 1:**
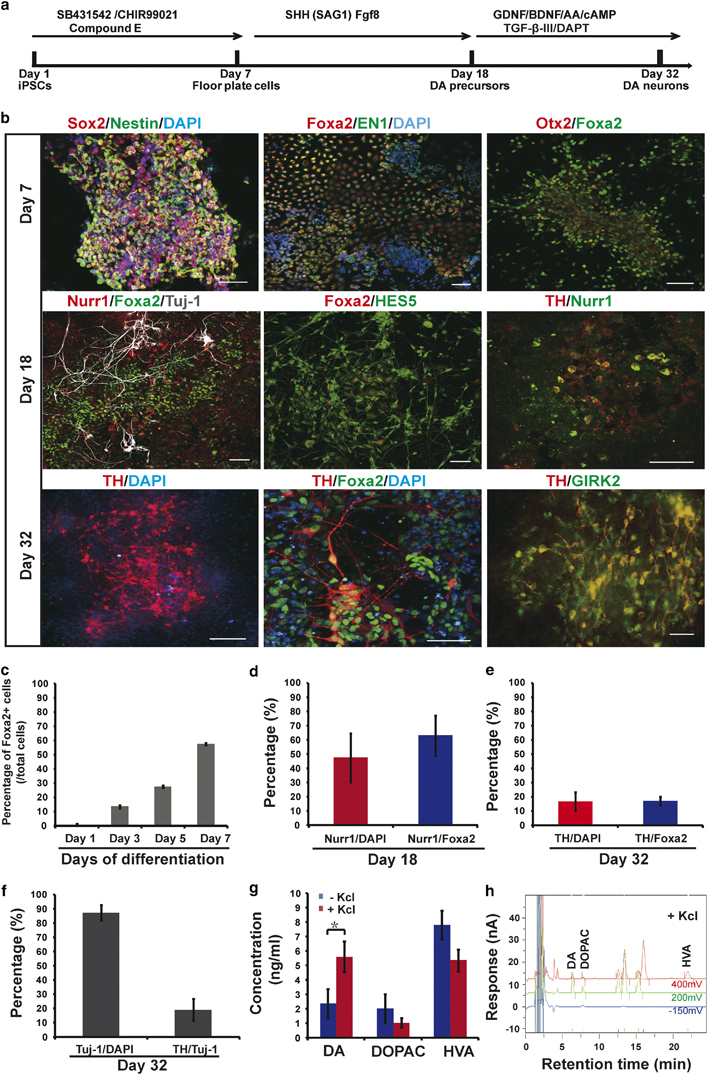
Differentiation of monkey induced pluripotent stem cells (iPSCs) to dopaminergic neurons. (**a**) Schematic representation of the differentiation procedure. (**b**) Immunostaining of differentiated cells on Days 7, 18, and 32. Scale bars, 50 μm. (**c**) Percentages of Foxa2+ cells on Days 1, 3, 5, and 7 (*n*=4). (**d**) Percentages of Nurr1+ cells on Day 18 (*n*=3). (**e**) Percentages of TH+ cells on Day 32 (*n*=3). (**f**) Fraction of Tuj-1+ cells among total cells and TH+ cells among Tuj-1+ cells on Day 32 (*n*=3). (**g**, **h**) Dopamine (DA), 3,4-dihydroxyphenylacetic acid (DOPAC), and homovanillic acid (HVA) levels on Day 32 before and after KCl stimulation using high-performance liguid chromatography (HPLC) methods (*n*=3; *P<0.05). For all the figures presented in this study, error bars represent s.e.m.

A recent study reported that monolayer human iPSCs can be directed toward leukemia inhibitory factor (LIF)-dependent expandable primitive neural epithelium stem cells (p-NSCs) when treated with LIF, SB431452 (SB), CHIR99021 (CHIR), and Compound E (C-E) [16] for 7 days. We tested this strategy on the monkey iPSCs. Monkey iPSCs cultured as monolayer on feeder cells were treated with the above molecules (1:1 DMEM/F12: Neurobasal, supplemented with LIF, SB, CHIR, and C-E) for 3 or 7 days, and neural cells were readily detected (Supplementary Figure S2A). However, those cells could not be passaged in the medium without C-E (LIF, SB, and CHIR only) as reported with human cells [16]. C-E is an inhibitor of Notch signaling pathway. Addition of C-E in the first few days can accelerate the conversion of iPSCs to NSCs; however, C-E suppresses cell proliferation at the NSC stage, and therefore was removed at the expansion stage, as shown in study by Ding and collegues [16]. The gestation period for monkey (36 weeks) is shorter than that of human (40 weeks). Accordingly, with the current differentiation scheme, the time length required for differentiation of iPSCs to neural cells might be shorter for monkey cells than human cells (around 5 days). If this is true, the presence of C-E throughout the first 5 or 7 days of differentiation may account for the lack of proliferative NSCs. To test whether this is the case, we removed C-E in the initial differentiation stage (only with LIF, SB, and CHIR). However, still no expandable NSCs were observed (data not shown), suggesting that failure to obtain monkey proliferative NSCs may be the result of species difference. Addition of LIF in the initial stage was aimed at selecting LIF-responsive proliferative NSCs. Failure to detect NSCs led us to question whether it is necessary to add LIF in the initial stage. Without LIF, monkey iPSCs were still able to generate neural cells (data not shown). Therefore, we tailored the protocol and only used SB, CHIR, and C-E for the initial stage of differentiation. Foxa2 is a transcription factor that emerges early and persists throughout the process of DA neuron specification [17, 18]. It has been shown that CHIR is a critical factor for inducing Foxa2 and Limx1a expression [19]. Expression of Foxa2 may confer the cells the competency to respond well to the morphogens SHH and Fgf8 [17, 20]. Treatment with SB, CHIR, and C-E for 3, 5, or 7 days resulted in 13.6, 27.8 and 57.6% of Foxa2-positive cells, respectively (Figure 1c). Therefore, we chose 7 days as the time length for the first stage (Figures 1a and b). At the end of this stage, cells also expressed Nestin, Sox2, EN1, and Otx2 (Figure 1b, Day 7). SHH and Fgf8 are well known to drive the specification of DA neurons [21]. We used Fgf8, and SAG1, a small molecule that can activate SHH signaling [22], in the second stage for the further patterning of DA neurons. Nurr1 is a marker expressed by post-mitotic DA neurons, and its expression has been used to select appropriate stages of cells to balance cell survival and maturation/integration in a DA neuron transplantation study [23]. On day 18, Nurr1-positive cells constituted around 47.5% of the total cells. At this stage, some cells in culture were also positive for Foxa2 (91.0%, Supplementary Figure S2B) and HES5 (38.1%), and a small proportion positive for TH (8.4%) (Figure 1b, Day 18 and Figure 1d). Cells at this time point were chosen to test transplantation in non-obese diabetic-severe combined immunodeficient (NOD-SCID) mice. From day 18 onward, the culture was treated with glial cell line-derived neurotrophic factor (GDNF), brain derived neurotrophic factor (BDNF), ascorbic acid, cyclic adenosine monophosphate (cAMP), transforming growth factor (TGF)-beta III, and DAPT for generation of mature DA neurons. On day 32, about 17% of cultured cells expressed TH (Figure 1e), and about 15.6% of total cells were positive for both TH and Foxa2. About 62.0% of TH+ cells also stained positive for A9 region-specific marker GIRK2 (about 10.5% of total cells). The supernatant of cultures on day 32 with or without potassium chloride (KCl) stimulation was collected and tested for levels of dopamine and its metabolites 3,4-dihydroxyphenylacetic acid (DOPAC) and homovanillic acid (HVA) (Figures 1g and h). Dopamine secretion was detected in the culture and its expression increased with KCl stimulation.

### Engraftment of monkey iPSC-derived DA cells to immunodeficient mice

**Figure 2:**
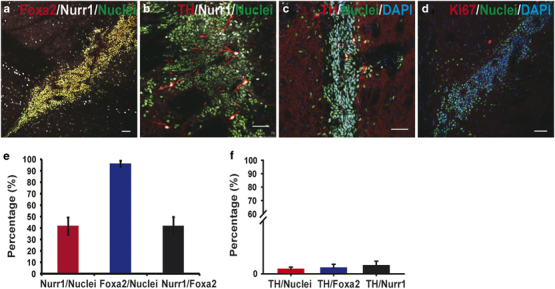
Transplantation of monkey induced pluripotent stem cell (iPSC)-derived dopamine (DA) cells into brains of immunodeficient mice. (**a**–**d**) Immunofluorescence staining of NOD-SCID mouse brain sections for TH, human/monkey nuclei, Nurr1, Foxa2, and Ki67 at 4 weeks after grafting. Scale bars, 50 μm. (**e**) Quantification of Nurr1+ and Foxa2+ cells in monkey iPSCs-derived grafts 4 weeks after transplantation into non-obese diabetic/severe combined immunodeficient (NOD-SCID) mouse brains (*n*=4). (**f**) Fractions of TH+ cells among Foxa2-, Nurr1-, and Nuclei-positive cells in monkey iPSCs-derived grafts 4 weeks after transplantation into NOD-SCID mouse brains (*n*=4).

One of the major concerns associated with iPSC-derived cells is the risk of tumor development. Next we tested the safety of the cells by transplanting day 18 culture into the striatum of naive immunodeficient mice. Four weeks after engraftment, the brains were sliced and analyzed by immunofluorescence staining. Human/monkey nuclei-specific antibodies were used to identify the surviving grafted cells. The fractions of TH-, Nurr1-, and Foxa2-positive cells were calculated. More than 95% of surviving cells at striatum were positive for Foxa2 (Figures 2a and e), and Nurr1- and TH-positive cells were about 44.8 and 0.8%, respectively (Figures 2b, c, e and f). No Ki67/monkey nuclei-double positive cells were found in the grafts (Figure 2d). The substantia nigra A9 DA neurons are relatively more vulnerable than other neuronal types [24–26], and accordingly, may experience more difficulties surviving the transplantation procedure and adapting to the *in vivo* environment. This may explain the discriminating cell death of TH+ neurons 4 weeks after engraftment into the immunodeficient mouse brain (0.8% TH+ cells), versus that of *in vitro* culture on Day 18 (8.4% TH+ cells).

### Autologous transplantation of iPSC-derived DA cells into the brain of a cynomolgus monkey PD model

**Figure 3:**
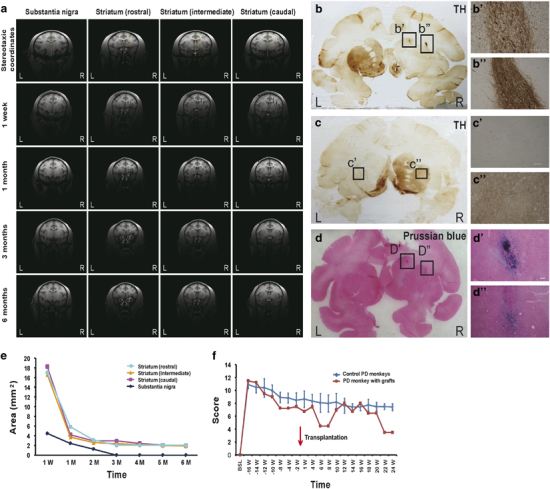
Autologous transplantation of monkey induced pluripotent stem cell (iPSC)-derived dopamine (DA) neurons and outcome analysis. (**a**) Magnetic resonance imaging (MRI)-guided stereotaxy and T2-weighted MRI at various time points after transplantation. MRI demonstrated that the cells had been accurately deposited into the target areas. (**b**, **c**) DAB staining for detection of TH+ cells and fibers (**b**, bʹ, bʹʹ: Parkinson's disease (PD) model with grafts; **c**,cʹ,cʹʹ: PD model without grafts) 6 months after transplantation. (**d**) Prussian blue staining to visualize transplanted cells in the lesioned side of striatum 6 months after transplantation. (**e**) The MRI signal areas of grafts over time. (**f**) The monkey engrafted with autologous iPSC-derived DA neurons showed behavioral recovery 6–8 weeks and 22–24 weeks after transplantation, whereas sham-grafted animals did not. Scale bars, **b**–**d**: 100 μm.

Four monkeys (4–9 years old) including the one from which the iPSCs were derived were subjected to 1-methyl-4-phenyl-1,2,3,6-tetrahydropyridine (MPTP) administration through unilateral carotid injection. Four months later, when the PD symptoms were stabilized, the iPSC donor monkey received autologous iPSC-derived DA neural cell transplantation, and the other three PD monkeys served as non-transplantation controls. We chose three transplantation sites at the rostral, intermediate, and caudal levels of the caudate nucleus and putamen, respectively, as well as one site just above the layer of substantia nigra, in the MPTP-damaged hemisphere (Figure 3a and Supplementary Table S1). According to the results of NOD-SCID mice transplantation using cells at Day 18, the fraction of TH- and Nurr1-positive cells were only 0.8 and 44.8%, respectively (Figures 2e and f), not as much as we had expected. We speculated that including cells a few days beyond the patterning stage (stage 2) may help improve outcome. Therefore, we mixed cells at day 18 and cells at day 22 (4 days in stage 3 in maturation medium) at a ratio of 1:1. For tracking purposes, the cells were labeled with green fluorescence protein at iPSC stage (Supplementary Figures S3A and B) and iron nano-particles before transplantation. The iron particles show signals in magnetic resonance imaging (MRI). The areas of positive signals were measured 1 week before and every month after transplantation until the end of the experiments (6 months after engraftment). The number of surviving cells at striatum as indicated by the signal areas decreased over time and stabilized at around 3 months post-transplantation (Figure 3e). The signals right above substantia nigra disappeared at 3 months, suggesting that cells deposited at this site may have died by this time.

At the end of the experiment (6 months after engraftment), the four PD monkeys including three controls were analyzed by brain slice staining. Immunohistochemical staining with DAB as a substrate revealed that TH-positive signals were almost absent in the striatum of the lesioned side (Figures 3b and c), except at the transplantation sites (Figures 3bʹ and bʹʹ), where intensive TH signals were detected.

The iron particles exhibited blue color following Prussian blue staining, as shown in Figure 3d, confirming that the grafted cells had been deposited at the target areas, which was in agreement with the MRI results. No Prussian blue positive signals were detected at the layer above substantia nigra, suggesting that the cells deposited at this site may have died.

The symptoms of the PD monkeys normally stabilize 3-4 months after model establishment. In the current study, the three control PD monkeys that received no grafts all remained stable from week 0 (time of transplantation for the experiment group) through week 24 (end of experiment) with scores around 8–10 (moderate PD state). However, the PD monkey that had received autologous DA neural graft showed a remarkable recovery between 6 and 8 weeks post transplantation. Yet the scores bumped up back to the control level from 8 weeks post transplantation, until 22 weeks post transplantation, when the symptoms were ameliorated again, with the score reduced from around 8 to around 4 (Figure 3f and Supplementary Movie S1).

**Figure 4:**
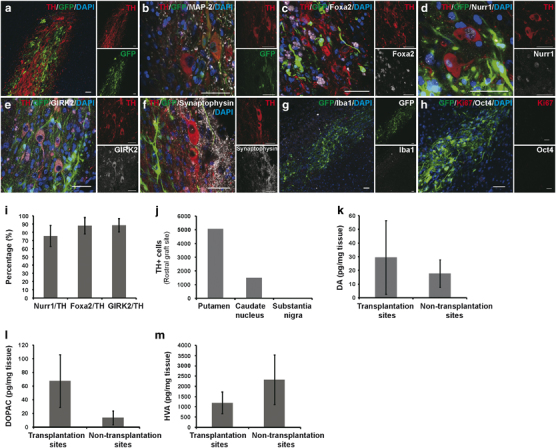
Graft analysis. (**a**, **b**) Immunofluorescence staining for green fluorescent protein (GFP)- and TH-double positive cells in grafts 6 months after transplantation. (**c**-**e**) Immunofluorescence staining for detection of TH+ cells that co-expressed Foxa2 (**c**), Nurr1 (**d**) and GIRK2 (**e**), A9 region-specific marker, in grafts 6 months after transplantation. (**f**) Immunofluorescence staining for detection of TH+ cells that co-expressed Synaptophysin in grafts 6 months after transplantation. (**g**,**h**) Immunofluorescence staining for Iba1, Oct4, and Ki67 in grafts 6 months after transplantation. (**i**) Fractions of TH+ grafted cells that co-expressed Nurr1, Foxa2, or GIRK2 (at least 100 cells were analyzed). (**j**) Quantification of surviving TH+ cells in grafts at rostral striatum. (**k–** **m**) high-performance liquid chromatography (HPLC) analysis of dopamine (DA) and the metabolites (3,4-dihydroxyphenylacetic acid (DOPAC) and homovanillic acid (HVA)) at transplantation sites versus ipsilateral non-transplantation sites. Scale bars, **a–h**, 50 μm.

Immunofluorescence staining revealed a significant number of TH-positive cells derived from the grafts deposited at the striatum (Figures 4a and b). About 88.0 and 75.2% of the TH-positive cells co-expressed Foxa2 and Nurr1, respectively (Figures 4c, d and i). In addition, about 88.5% of the TH-positive cells were also positive for GIRK2, an A9 DA neuron marker specifically expressed at the substantia nigra pars compacta (Figures 4e and i). Synaptophysin staining revealed that graft-derived neurons may have established synaptic connections with host neurons (Figure 4f). No microglia activation was detected at the graft sites (Figure 4g) and the grafted cells were negative for Ki67 or Oct4 (Figure 4h), confirming the safety of the current strategy. Furthermore, no graft-derived serotonergic neurons were detected (Supplementary Figure S3C).

We counted all the graft-derived TH-positive cells at the rostral level of striatum in every third section, and estimated that there were around 5 100 and 1 500 (Figure 4j) TH-positive cells in the putamen and caudate nucleus, respectively, at the rostral level of striatum (the intermediate level of striatum slab was used for high-performance liquid chromatography (HPLC) analysis). A rough estimation of surviving TH-positive cells in the whole brain was around 19 800 ((5 100+1 500) x 3).

To measure the concentration of DA, DOPAC, and HVA at the intermediate level of striatum, we sampled the graft sites and areas distant from the grafts in the ipsilateral hemisphere and performed HPLC analysis. The concentrations of DA and DOPAC at the graft sites were 29 pg mg^−1^ tissue, and 68 pg mg^−1^ tissue, respectively, higher than those at the ipsilateral non-grafted areas, which were 17 and 12 ng mg^−1^ tissue for DA and DOPAC, respectively (Figures 4k and m, the differences were not statistically significant due to too few samples). However, the DA and DOPAC levels at the graft sites were still much lower than those at the contralateral intact hemisphere (Supplementary Figures S3D-l).

## Discussion

In this study, we differentiated monkey iPSCs to DA cells using a tailored method with a relatively high efficiency, and transplanted autologous DA cells into the brain of a monkey PD model without immunosuppression. No overgrowth of grafts was observed. Part of the grafted cells survived and became mature DA neurons; the monkey that had received grafts experienced a behavioral improvement compared with controls without transplantation. The current study provided a proof-of-principle that iPSC-derived DA neurons may offer an autologous cell source for treatment of PD monkey models.

One major concern with regard to pluripotent stem cell-based therapy was the risk to develop tumors. Even a small number of residual pluripotent cells existing in the culture may cause tumor development, which is by no means acceptable in clinical practice. Two methods may be used to reduce the risk. One is to employ a sorting step to get rid of the contaminating pluripotent cells; the other is to improve the robustness of the differentiation procedure to a level sufficient to drive all the starter cells to exit pluripotent state. In the clinical trials using embryonic stem (ES) cell-derived retina pigment epithelial cells to treat age-related macular diseases, the donor retina pigment epithelial cells were isolated with a purity of at least 99% and no overgrowth of the grafts was observed even after a medium-to-long-term follow-up time [27–29]. In our study, a straightforward protocol-treatment of iPSCs with SB, CHIR, and C-E for 7 days, followed by SAG1 and Fgf8 for 11 days, resulted in a culture that had lost tumorigenic capacity when transplanted into the striatum of immunodeficient mice; this suggests that the combination of small molecules SB, CHIR, and C-E is powerful enough to push almost all of the iPSCs to exit the pluripotent state, and more interestingly, with a tendency to differentiate toward a ventral mesencephalon fate, with around 57% of all cells positive for Foxa2 on Day 7 (Figure 1c). On Day 18, Nurr1+ and Foxa2+ cells constituted around 48 and 92% of total cells, respectively. At this stage (Figure 1d), applying an additional sorting step may help to further enhance the purity of the cells. Future studies are needed to identify which surface molecule(s) can best match Nurr1 expression.

With regard to specification of DA neurons from iPSCs, different protocols have been reported [30–35], which include embryoid body differentiation method, co-culture with stroma cells, and dual SMAD inhibition on monolayer ES/iPS cells. These methods normally require at least 6 weeks of differentiation and yield about 5–10% TH-positive neurons and even less percentage of TH/Foxa2-double positive midbrain DA neurons. Relatively, our method takes shorter period of time (around 5 weeks to obtain mature DA neurons and 3 weeks to get transplantable DA precursor cells), requires fewer factors (only CHIR and SB in the first 7 days to drive iPSCs to floor plate cells), and gives rise to a higher efficiency (17% of TH-positive cells and 15% TH/Foxa2-double positive midbrain DA neurons). In future studies, we will investigate whether human iPSCs, employing similar method, can be efficiently differentiated to DA neurons that are safe and efficacious for cell therapy of PD.

In this study, only one cynomolgus monkey received autologous iPSC-DA cell transplantation, mainly due to the difficulty to obtain SFV-negative animals. More than 99% of cynomolgus monkeys are positive for SFV. Although the SFV-positive monkeys remain asymptomatic, the primary cultures derived from them normally require anti-viral treatment to be healthy; however, the antiviral treatment interferes with virus-mediated reprogramming process. We are currently testing the episomal methods (virus free) to derive iPSCs from monkey MSCs and peripheral blood cells. If it works, we will employ a larger cohort of animals in the next study to test the safety and efficacy of such strategies.

The engrafted cells mainly stayed in the target areas in striatum and extended processes beyond the boundary of the bolus (Figure 3b). Synaptophysin staining revealed that synaptic connections may have been established between the engrafted and endogenous neurons (Figure 4f). In our study, around 19 800 DA neurons survived in the striatum of the transplanted hemisphere, and the motor functions were improved in certain time windows post transplantation. Compared with the three control PD models that had received no grafts, the monkey that had received autologous iPSC-DA neurons experienced motor function recovery at 6–8 weeks and from 22 weeks onward to the end of the experiment (24 weeks post-transplantation, Figure 3f). Although we could not exclude the possibility that this change in motor function was due to the fluctuation of behavioral performance, this possibility was slim. The PD model used in the study was relatively stable, as we can see from the three control animals (Figure 3f and Supplementary Figure S3J). The model animals may experience certain degree of spontaneous recovery after MPTP administration, for example, from score 11 to 8 in 6 months as observed for the three control animals in our study. Nevertheless, this kind of spontaneous recovery went smoothly over time, and normally within certain range, not like what we had observed in the engrafted animal that showed a big drop from 11–12 to 4–5 during a period of 6 months; this drop in score took place relatively fast, from 7.5 in week 4 to 4.5 in week 6, and from 6.5 in week 20 to 3.5 in week 22. Given the degree and temporal pattern of changes, it is more likely that the behavioral improvement was due to DA cell transplantation.

It remains elusive as to what caused the short period of recovery from week 6 to 8, followed by a bump up of the score. During the first month after transplantation, a majority of grafts had died, as could be seen from the reduction in Feridex signal areas (Figure 3e). From week 6 to 8, the surviving cells were probably in the middle of assuming dynamic changes, such as differentiation and integration into the existing neuronal network. These dynamic changes of grafts might cause behavioral changes accordingly, which could be one speculative reason for the improved motor function from week 6 to 8. As to the behavioral recovery starting from week 22, it could be, optimistically, the start of a stable long-term improvement period, or, just the start of a temporal recovery period, similar to what happened during week 6–8. Six months after transplantation, the grafted cells may have gone through the surviving, differentiation, and integration processes, and remained relatively stable. During the preparation of this manuscript, Hallett *et al*. [36] reported a similar study in *Cell Stem Cell*, in which autologous transplantation of iPSC-derived DA cells in a cynomolgus PD model led to substantial DA neuron survival and behavioral improvement 2 years following engraftment. The one cynomolgus monkey that showed behavioral recovery had around 13 000 surviving TH+ graft-derived neurons, similar to that in our study (around 19 000 DA neurons). Moreover, the authors suggested that 13 000 may be the number of midbrain DA neurons needed to reach the threshold of functional improvement. Interestingly, the motor function started to improve at 6 months post transplantation, similar to the observation in our study, and reached a peak performance score at 12 months. It would be interesting to investigate the long-term transplantation result using our method of differentiation, and future studies are needed to test this.

In addition to iPSCs, induced DA (iDA) neurons and induced neural stem cells (iNSCs) are other potential sources that can offer autologous DA neurons. Our lab and others have reported generation of induced DA and iNSCs that are of utility for PD modeling and treatment [37–46]. iNSCs also possess the expandability and certain degree of plasticity for DA neuron specification. It would be interesting to test the safety and efficacy of iNSCs in large animal PD models in the future.

Taken together, in the current study, we examined the safety and efficacy of autologous iPSC-derived DA cells for the treatment of PD in a nonhuman primate model. The PD monkey that had received autologous grafts did not develop tumors and showed behavioral improvement in certain time frames. This case study warrants future studies employing a larger cohort of animals to confirm the safety and efficacy of such therapeutic strategies.

## Materials and Methods

### Subjects and ethics statement

Three adult male (4–9 years old) and one adult female (6 years old) cynomolgus monkeys, with body weights ranging from 3.7 (female) to 6.8 (male) kg, were supplied by Wincon TheraCells Biotechnologies (Guangxi, China), which is an AAALAC-accredited nonhuman primate research facility. The experiment protocol was approved by the Institutional Animal Care and Use Committee (IACUC), and the methods were carried out in accordance with the approved guidelines.

### Differentiation of iPSCs to DA cells

iPSCs were infected with lentiviral vectors expressing GFP (FUGW, Addgene, Cambridge, MA, USA) for tracking purposes. On Day 1 of differentiation, hES-conditioned medium was changed to differentiation Medium I containing Neurobasal A/DMEM-F12 1:1, with or without 10 ng ml^−1^ human LIF, 3 μM CHIR99021, and 2 μM SB431542. On Day 8, cells were treated with Medium II containing Neurobasal A/DMEM-F12 1:1, 10 μM SAG1, and 100 ng ml^−1^ Fgf8 for 10 days. On Day 19, medium was changed to Medium III consisting of Neurobasal A/DMEM-F12 1:1, 0.2 mM ascorbic Acid, 0.5 mM cAMP, 1ng ml^−1^ TGF-beta III, 10 ng ml^−1^ GDNF, 10 ng ml^−1^ BDNF, and 10 μM DAPT.

### Engraftment of monkey iPSC-derived DA cells into NOD-SCID mouse brains

Monkey iPSCs were differentiated according to Figure 1a, and the cells on Day 18 were collected and transplanted into the striatum of NOD-SCID mice (male, 6 weeks old). Four weeks later, the mice were killed and brains taken out for analysis.

### Immunostaining

Immunostaining was performed as previously described [47]. The antibody information was listed in Supplementary Table S2.

### Establishment of PD monkey model

After the collection of baseline data, each monkey received a single unilateral intracarotid artery injection of 3 mg of MPTP-HCl (Sigma, St. Louis, MO, USA) in 30 ml of saline (rate: 1 ml min^−1^) to induce a stable model of hemi-parkinsonism as previously described [48].

### MRI and cell transplantation

MRI was performed as previously described [47]. Before transplantation, the GFP+ cells were double-labeled with Feridex IV (100 mg ml^−1^, Advanced Magnetics, Rochester, MN, USA) according to the manufacturer’s instruction. The cells were suspended at 1×10^8^ cells ml^−1^ in HBSS buffer with 5 g l^−1^ D-glucose, 100 ng ml^−1^ BDNF, 100 ng ml^−1^ GDNF, and 0.2 mM ascorbic Acid, and injected into target areas guided by MRI, as previously described [47]. The coordinates of graft sites were shown in Supplementary Table S1. The three control PD monkey models were injected with vehicle buffer (HBSS with 5 g l^−1^ D-glucose, 100 ng ml^−1^ BDNF, 100 ng ml^−1^ GDNF, and 0.2 mM ascorbic acid) using the same coordinates.

### Behavioral analysis

Monkeys were evaluated using a clinical rating scale as previously described [49]. In brief, animals were videotaped and evaluated blindly by scoring from 0 to 3 points in the following categories: bradykinesia (0–3), upper limb rigidity (0–3), lower limb rigidity (0–3), upper limb tremor (0–3), lower limb tremor (0–3), food pickup with affected hand (0–3), body posture (0–2), and balance (0–2). The minimal score was 0 and the maximum disability score was 22. A score of 8 corresponds to a moderate parkinsonian state.

### Tissue preparation and histology

Six months after transplantation, the monkeys were killed and brains were taken out and cut into slabs. For HPLC, areas of interest were punched out and stored at −80 °C. For histochemical staining, slabs were fixed and sectioned. Prussian blue staining was used to detect iron within the transplanted cells. Antibodies used were listed in Supplementary Table S2. To quantify TH+ neurons, one out of every three serial sections (40 μm) throughout the graft sites were stained and counted at ×20 magnification.

### HPLC

DA, HVA, and DOPAC levels in brain tissues and culture supernatant were measured as previously described [47]. As to cultured cells, iPSC-derived neurons on day 32 were incubated in high potassium-HBSS medium with nomifensine (10 μM, Sigma) and pargyline (50 μM, Sigma) for 45 min at 37 °C. One hudred and eighty microliters of medium was then transferred to eppendorf tubes with 20 μl 1 N perchloric acid (Sigma) and stored at −20 until use.
