# Supplementary material for: Autologous iPSC-derived dopamine neuron transplantation in a nonhuman primate Parkinson’s disease model
Source: Cell Discov. 2015 May 26;1:15012–. doi: 10.1038/celldisc.2015.12 (PMC4860772; doi:10.1038/celldisc.2015.12)
Supplement: Supplementary Information [file celldisc201512-s1.doc]

## Supplementary Materials

## Autologous iPSC-derived dopamine neuron transplantation in a nonhuman primate Parkinson’s disease model

Shuyan Wang^1,2,3, †^, Chunlin Zou^4^, ^†^, Linlin Fu^1^, Bin Wang^1^, Jing An^1^, Gongru Song^1^, Jianyu Wu^1^, Xihe Tang^1^, Mo Li^1^, Jian Zhang^4^, Feng Yue^5^, Chengyun Zheng^6^, Piu Chan^3,5^, Y. Alex Zhang^1^, Zhiguo Chen^1,2,3,*^

^1^ Cell Therapy Center, Beijing Institute of Geriatrics, Xuanwu Hospital, Capital Medical University, and Key Laboratory of Neurodegeneration, Ministry of Education, Beijing 100053, China

^2^ Center of Neural Injury and Repair, Beijing Institute for Brain Disorders, Beijing, 100069, China

^3^ Center of Parkinson's Disease, Beijing Institute for Brain Disorders, Beijing, 100069, China

^4^ Center for Translational Medicine, Guangxi Medical University, Nanning, 530021, Guangxi Province, China

^5^ Department of Neurobiology, Beijing Institute of Geriatrics, Xuanwu Hosptial, Capital Medical University, Beijing, 100053, China

^6^ Department of Hematology, Second Hospital of Shandong University, Jinan, 250033, Shandong Province, China

^†^ Equal contribution

*Correspondence and requests for materials should be addressed to Zhiguo Chen ([chenzhiguo@gmail.com](mailto:chenzhiguo@gmail.com)).

**Supplementary materials and methods**

*MSC isolation and expansion*

Bone marrow (BM) sample was harvested from an adult cynomolgus monkey. Mononuclear cells (MNCs) were isolated from heparinized BM aspirates using Ficoll gradient and subsequently plated into a single T-75 flask at a density of 2.5×10^7^ MNCs/flask. The floating cells were removed after 48 hours and the medium was changed every 3-4 days thereafter. By day 11-12 the primary cultures reached approximately 70-80% of confluence, and about 2-3 million cells were harvested in total, and replated at a density of 6,000 cells/cm^2^ henceforth.

*Generation of iPSCs*

iPSCs were generated using a protocol developed by Yamanaka and colleagues. Briefly, MSCs were plated at a density of 1×10^4^ cells /cm^2^ prior to the day of infection. 24 hrs later, virus cocktail (Oct4, Sox2, Klf4, c-Myc) and 6 μg/ml polybrene in fibroblast culture medium were added. Mitomycin-treated CF1 feeders were pre-plated at a density of 2×10^6^ per 10 cm^2^ plate on the 5^th^ day of infection. On day 6 post-infection, cells were treated with 0.05% Trypsin-EDTA and replated onto the CF1 feeders. After overnight incubation, the fibroblast culture medium was changed to human ES medium, and thereafter, medium was changed every other day or every day, as required. Human ES like colonies appeared after 14-21 days post infection. Colonies were picked manually and transferred onto MEF-coated plates and cultured in hES medium for expansion. For passaging, the confluent iPSCs were incubated with Collagenase Ⅳ at 37 ℃ for 20 min, washed with PBS, and scraped off from the plate with the tip of a 2 ml pipette. Then the cells were collected into centrifuge tubes, spun down, and re-suspended with fresh medium, and spit at a ratio of 1:6.

*Immunostaining of undifferentiated monkey iPSCs*

To characterize clones by immunostaining for various markers, cultured monkey iPSCs were fixed and stained for TRA-1-60, SSEA-4, NANOG, and OCT4 (Table S2). After a brief wash with PBS, Alexa555 conjugated goat anti-rabbit or anti-mouse secondary antibodies (1:500, Invitrogen) were used for one-hour incubation to visualize the cells together with DAPI nuclear staining. In order to visualize the alkaline phosphatase (AP) activity, iPSCs were fixed and stained with Sigma’s FAST BCIP/NBT (B5655, Sigma). The AP assay was performed per the manufacturer’s instructions.

*Embryoid body (EB) formation and immunostaining*

EBs were formed using differentiation medium (expansion medium withdrawing bFgf and replacing 20% KSR with 20% FBS). iPSCs (near confluent) from 2 wells of a 6-well plate were used for EB formation test and cultured in one well of ultra-low attachment 6-well plate. After 8 days, EBs were transferred to gelatin-coated 24-well plates to allow attachment for additional 2 days. For immunostaining of EBs, the following primary antibodies were used: Tuj-1, α-SMA, and AFP (Table S2). Alexa555-conjugated donkey anti-mouse or anti-rabbit secondary antibodies (1:500, Invitrogen) were used for visualization.

*Teratoma assay*

The use of immunodeficient mice for teratoma assay was approved by the Animal Care and Use Committee at Xuanwu Hospital Capital Medical University. Three to five million iPSCs were harvested by Collagenase IV (Sigma) digestion, washed with PBS and re-suspended in 200 μl diluted (1:1) Matrigel solution (BD). Cells were injected intra-muscularly into NOD-SCID immunodeficient mice. Teratomas were excised 5 weeks after injection. After sectioning, slides containing various regions of teratomas were stained by H&E method.

*Karyotype analysis*

For karyotype analysis, the parental MSCs and iPSCs were treated with 20 μg/ml Colcemid (Sigma) at 37°C for 30 min, detached by 0.125% Trypsin (Life technologies) and then treated with 0.075 M KCl (Sigma). The cells were stained with freshly prepared 4% Giemsa stain in Gurr buffer (both from Life technologies) and examined under light microscope. The karyotype was printed by assigning a number to each sister chromatid pair.

**Supplementary Figures**

**
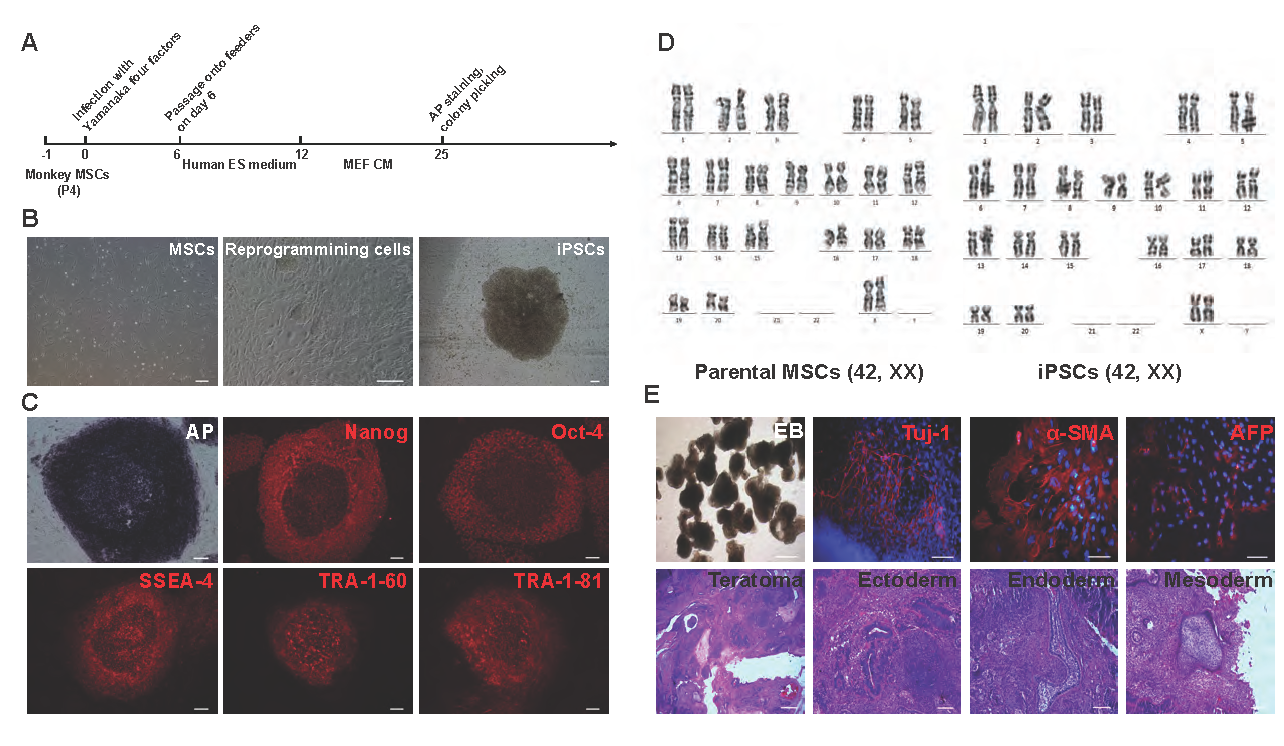
**

**Figure S1. Generation of induced pluripotent stem cells (iPSCs) from MSCs of an adult cynomolgus monkey.**

(A) Schematic representation for iPSC generation procedure.

(B) MSCs before reprogramming (left), emerging colonies of iPSCs on MEF feeders (middle), and an established iPSC clone (right). Scale bars, 200 μm.

(C) The iPSC clone expressed pluripotent markers. Scale bars, 100 μm.

(D) The iPSCs showed a normal female cynomolgus monkey karyotype (42, XX) after 10 passages.

(E) The iPSCs could form embryoid bodies (EBs) and be differentiated to cells of the three germ layers positive for Tuj-1 (ectoderm), α-SMA (mesoderm), and AFP (endoderm). When injected into NOD-SCID mice, iPSCs developed into teratomas, consisting of tissue types from endoderm (respiratory epithelium), mesoderm (cartilage), and ectoderm (neural tube). Scale bars, EB and Teratoma: 500 μm; others, 100 μm.


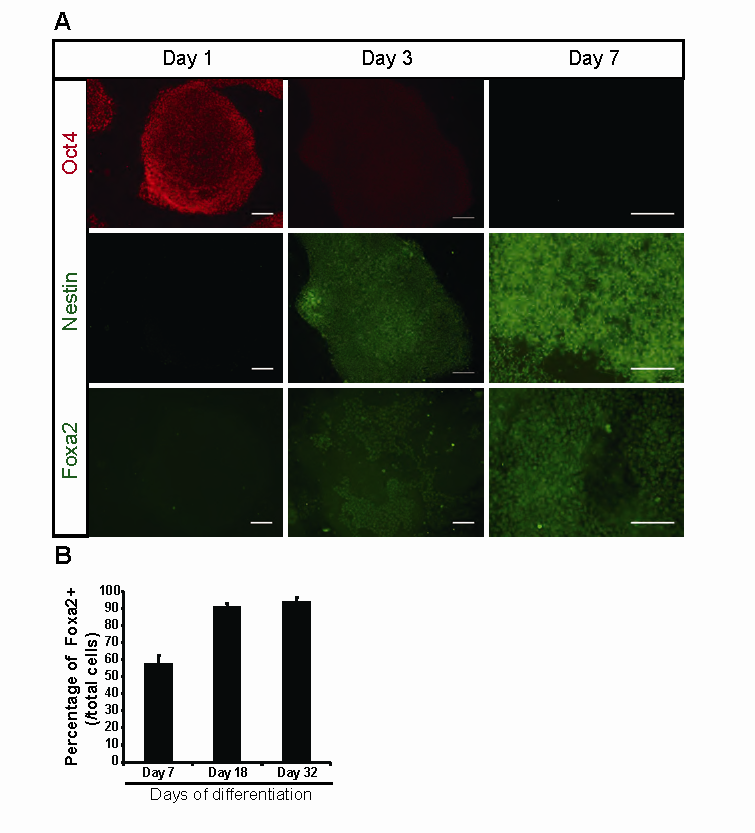


**Figure S2. Differentiation of monkey iPSCs to dopamine lineage cells *in vitro*.**

(A) Immunoﬂuorescence staining of differentiated cells on Days 1, 3 and 7, for Oct4 (upper), Nestin (middle), and Foxa2 (lower panel). Scale bar, 100 μm.

(B) Percentages of Foxa2+ cells at Days 7, 18 and 32 (n=4).

**
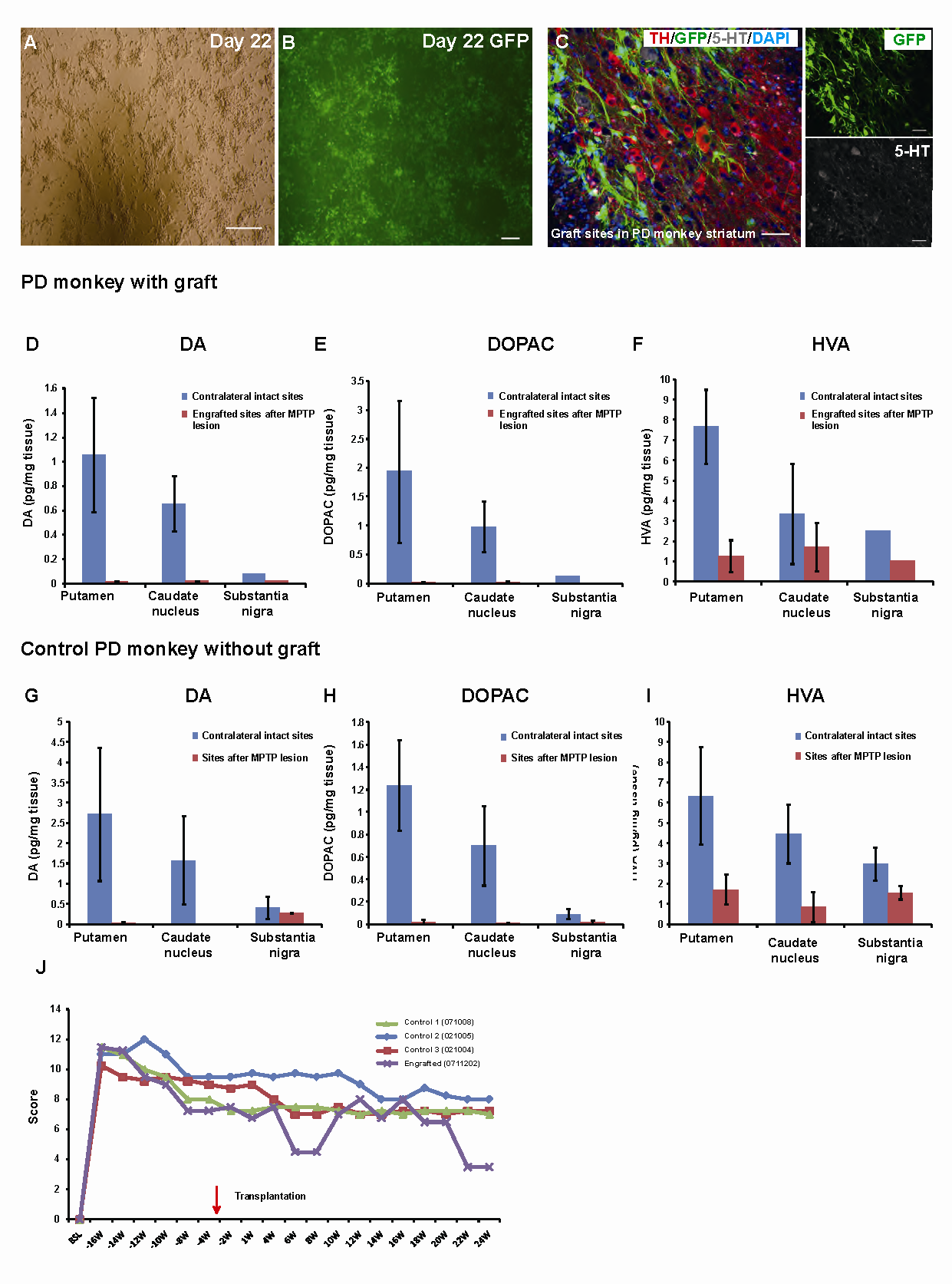
**

**Figure S3. Autologous transplantation into a monkey PD model and outcome analysis.**

(A, B) Monkey iPSCs-derived DA cells were labeled with GFP. Scale bar, 100 μm.

(C) Immunofluorescence staining of 5-HT, TH, and GFP in the grafts 6 months after autologous transplantation into the PD monkey. No GFP-labeled engrafted cells co-expressed 5-HT. Scale bar, 50 μm.

(D-I) DA, DOPAC, and HVA levels at the engrafted sites and the corresponding sites in the contralateral intact hemisphere, in the engrafted PD model and control PD model without transplantation.

(J) Behavioral results of the four monkeys employed in the study.

**Supplementary Tables**

**Table S1. Intracerebral positions of the catheter needle tip.**

|  | Subject |  | 0711202 (ID) |
| --- | --- | --- | --- |
| Site/Volume |  | Three dimensions |
|  |  | AP | 4 |
| Rostral caudate (10 μl) |  | Lat | 59.51 |
|  |  | V | 53.44 |
|  |  | AP | 10 |
| Intermediate caudate (10 μl) |  | Lat | 58.87 |
|  |  | V | 58.49 |
|  |  | AP | 14 |
| Caudal caudate (10 μl) |  | Lat | 58.66 |
|  |  | V | 57.64 |
|  |  | AP | 4 |
| Rostral putamen (10 μl) |  | Lat | 56.09 |
|  |  | V | 53.34 |
|  |  | AP | 10 |
| Intermediate putamen (10 μl) |  | Lat | 54.59 |
|  |  | V | 52.19 |
|  |  | AP | 14 |
| Caudal putamen (10 μl) |  | Lat | 53.31 |
|  |  | V | 53.04 |
|  |  | AP | 18 |
| Substantia nigra (5 μl) |  | Lat | 60.58 |
|  |  | V | 43.01 |

**Table S2. Antibody information.**

| Name | Cat. | Company | Host | Dilution factor |
| --- | --- | --- | --- | --- |
| Foxa2 (HNF-3β ) | SC-6554 | Santa Cruz | goat | 200 |
| HES-5 | AB5708 | Millipore | rabbit | 400 |
| Nurr1 | SC-990 | santa cruz | rabbit | 200 |
| GIRK2 | AB30738 | Abcam | rabbit | 100 |
| TH | AB152 | millipore | rabbit | 500 |
| TH | AB1542 | Millipore | sheep | 500 |
| GFAP | ZA-0117 | ZSGB-Bio | rabbit | 100 |
| Otx2 | BAF1979 | R&D | goat | 500 |
| Iba1 | Ab107159 | Abcam | goat | 1000 |
| 5-HT (serotonin) | ZA-0231 | ZSGB-Bio | rabbit | 200 |
| Nuclei | MAB1281 | Millipore | mouse | 500 |
| Tuj-1(beta-tubulin-III） | MAB1637 | Millipore | mouse | 400 |
| Synaptophysin | S5867 | Sigma | mouse | 500 |
| MAP-2 | SC-32791 | Santa Cruz | mouse | 500 |
| Ki67 | NCL-L-Ki67-MM1 | Novocastra | mouse | 200 |
| Nestin | AB5922 | Millipore | rabbit | 500 |
| Sox2 | 48-1400 | Life Technologies | rabbit | 500 |
| Oct4 | SC-5279 | Santa Cruz | mouse | 200 |
| EN1 | SC-66876 | Santa Cruz | rabbit | 200 |
| GFP | SC-9996 | Santa Cruz | mouse | 200 |
| GFP | A11122 | Life Technologies | rabbit | 200 |
| TRA-1-60 | MAB4360 | Millipore | mouse | 300 |
| SSEA-4 | MAB4304 | Millipore | mouse | 100 |
| NANOG | 500-P236 | Peprotech | rabbit | 300 |
| OCT4 | #2750 | Cell Signaling Technology | rabbit | 400 |
| Tuj-1(beta-tubulin-III) | T8578 | Sigma | mouse | 1000 |
| AFP | Ab114028 | Abcam | rabbit | 500 |
| α-SMA | CBL171 | Millipore | mouse | 500 |

**Supplementary movie 1**

Video clips showing the behavior of the monkey prior to MPTP challenge, after MPTP challenge and before transplantation, and six months after autologous iPSC-derived DA cell transplantation.
